# rpoB Gene mutations in rifampin-resistant Mycobacterium tuberculosis identified by polymerase chain reaction single-stranded conformational polymorphism.

**DOI:** 10.3201/eid0706.010615

**Published:** 2001

**Authors:** M Bobadilla-del-Valle, A Ponce-de-Leon, C Arenas-Huertero, G Vargas-Alarcon, M Kato-Maeda, P M Small, P Couary, G M Ruiz-Palacios, J Sifuentes-Osornio

**Affiliations:** Laboratory of Clinical Microbiology, Instituto Nacional de Ciencas Médicas y Nutritión Salvador Zubiran, Mexico City, Mexico.

## Abstract

The use of polymerase chain reaction-single-stranded conformational polymorphism (PCR-SSCP) to study rpoB gene mutations in rifampin-resistant (RIFr) Mycobacterium tuberculosis has yielded contradictory results. To determine the sensitivity of this method, we analyzed 35 RIFr strains and 11 rifampin-susceptible (RIFs) strains, using the DNA sequencing of the core region of rpoB for comparison. Of the RIFr, 24 had a PCR-SSCP pattern identical to that of H37Rv; the other 11 had four different patterns. The 11 RIFs had PCR-SSCP patterns identical to that of H37Rv. The sensitivity of the assay was 31.4%; its specificity was 100%. We observed a strong correlation between the degree of resistance and the type of mutation.


### *rpo*B Gene Mutations in Rifampin-Resistant *Mycobacterium tuberculosis* Identified by Polymerase Chain Reaction Single-Stranded Conformational Polymorphism

**Miriam Bobadilla-del-Valle,* Alfredo Ponce-de-Leon,* Catalina Arenas-Huertero,* Gilberto Vargas-Alarcon,† Midori Kato-Maeda,* Peter M. Small,‡ Patricia Couary,* Guillermo M. Ruiz-Palacios,* and Jose Sifuentes-Osornio***

*Instituto Nacional de Ciencias Médicas y Nutrición Salvador Zubiran, Mexico City, Mexico; †Instituto Nacional de Cardiología Ignacio Chavez and Universidad Panamericana, Mexico City, Mexico; and ‡Stanford University Medical Center, Stanford, California, USA

The use of polymerase chain reaction-single-stranded conformational polymorphism (PCR-SSCP) to study *rpo*B gene mutations in rifampin-resistant (RIFr) *Mycobacterium tuberculosis* has yielded contradictory results. To determine the sensitivity of this method, we analyzed 35 RIFr strains and 11 rifampin-susceptible (RIFs) strains, using the DNA sequencing of the core region of *rpo*B for comparison. Of the RIFr, 24 had a PCR-SSCP pattern identical to that of H37Rv; the other 11 had four different patterns. The 11 RIFs had PCR-SSCP patterns identical to that of H37Rv. The sensitivity of the assay was 31.4%; its specificity was 100%. We observed a strong correlation between the degree of resistance and the type of mutation.

In the developed world, tuberculosis (TB), once considered to have been essentially eliminated, has rebounded and is increasingly caused by drug-resistant strains. In developing countries, however, TB has been an unrelenting scourge. Increasing international travel and migration contribute to its widespread dissemination. Consequently, in 1993, the World Health Organization declared TB to be a global emergency (1).

Drug-resistant TB is a widespread phenomenon, with primary isoniazid-resistance rates as high as 32% and primary multidrug resistance close to 15% in the former Soviet Union. In Latin America, primary resistance to isoniazid varies from 1% in Uruguay to 20% in the Dominican Republic, and primary multidrug resistance is as high as 7% in the Dominican Republic and 5% in Argentina (2). In 1995, we reported increasing resistance rates to isoniazid and rifampin, four times higher than previously reported rates for Mexico (3). Since then, several studies have addressed this issue in different settings: urban, semi-urban, and rural areas. The common finding has been a high rate of primary resistance to isoniazid and to the combination of isoniazid and rifampin (4,5). In 2000, a collaborative effort between the Centers for Disease Control and Prevention and the Mexican TB control program reported an 11% rate of primary isoniazid resistance and 2% of primary multidrug resistance (6).

From the public health perspective, the impact of resistance on disease and death has recently been emphasized (7) in settings where HIV is highly prevalent. However, its impact is also high in semi-urban settings without the influence of HIV infection (8). Thus, reliable methods are urgently needed to rapidly detect resistance, particularly to rifampin (a marker for multidrug resistance), without cumbersome traditional methods or use of radioactivity (9).

Several techniques use polymerase chain reaction (PCR)-based strategies to rapidly detect mutations known to confer resistance. One such method is single-stranded conformational polymorphism (SSCP) analysis, which involves amplification by PCR of a segment of the gene encoding for the specific drug target and comparison of PCR products of drug-sensitive and drug- resistant strains by SSCP, in which mutations usually result in an altered pattern (9,10). This technique is relatively simple and was promising initially, but recent studies have questioned its sensitivity and specificity (10). We investigated the usefulness of PCR-SSCP to detect mutations in the *rpo*B gene of *Mycobacterium tuberculosis* strains with a wide range of rifampin resistance and whether specific mutations in this gene are associated with degree of rifampin resistance.

### Methods

### Clinical Isolates

Forty-six clinical isolates of *M. tuberculosis* were included in this study; all isolates were recovered from sputum samples of patients from Mexico City and were fully characterized by conventional methods (11). All strains were resistant to at least one primary antituberculosis agent (isoniazid 0.1 µg/mL, rifampin 2 µg/mL, streptomycin 6 µg/mL,

Address for correspondence: Jose Sifuentes-Osornio, Departamento de Infectología, Instituto Nacional de Ciencias Médicas y Nutrición Salvador Zubiran, Vasco de Quiroga 15, Tlalpan, México 14000 D.F. México; fax: (525) 513-3945; e-mail: jso@quetzal.innsz.mx or ethambutol 7.5 µg/mL). Thirty-five strains were rifampin resistant (RIFr), and 11 were rifampin sensitive (RIFs). MICs to the primary antituberculosis drugs were determined by the radiometric method (Becton Dickinson, Cockeysville, MD) (12).

### PCR Amplification

Chromosomal DNA was extracted by conventional methods (13). A 157-bp fragment of the *rpo*B gene was amplified by PCR with primers Tb8 (5'TGCACGTCGCGG ACCTCCA3') and Tb9 (5'TCGCCGCGATCAAGGAGT3'). PCR was carried out in 50 µL of a reaction mixture containing 50 mM KCl, 10 mM Tris (pH 8.0), 1.5 mM $MgCl_2$, 100 µM of deoxynucleoside triphosphates (dNTPs), 1U *Taq* polymerase, 10 pmoles of each set of primers, and 10 ng of chromosomal DNA. Samples were then subjected to one cycle at 94°C for 5 min, followed by 40 cycles at 94°C for 1 min, 55°C for 1 min, 72°C for 1 min, and a final cycle at 72°C for 8 min to complete the elongation of the PCR intermediate products. PCR products were then run on 2% agarose gels and examined for the presence of the 157-bp band after ethidium bromide staining.

### Screening of SSCP-PCR Products

The SSCP of PCR products was analyzed by electrophoresis with 12% acrylamide gels. In brief, 25 µL of the amplified product was diluted with 100 µL of buffer (0.1% sodium dodecyl sulfate, EDTA 10 mM); 3 µL of this dilution was mixed with 3 µL of loading buffer (95% formamide, 20 mM EDTA, and 0.05% each of bromophenol blue and xylene cyanol). The mixtures were boiled for 2 min, cooled in ice for 5 min, and then loaded on the gel at 40V for 10 h at room temperature. The gels were silver stained and allowed to dry. The drug-susceptible strain H37Rv was run side by side with the clinical isolates as a control for all experiments. Three different PCR products were analyzed five times each.

### DNA Sequencing

A 411-bp fragment of the *rpo*B gene, containing the sequence of the 157-bp *rpo*B fragment, was amplified by PCR using primers TR1 (5' TACGGTCGGCGAGCT GATCC3') and TR2 (5'TACGGCGTTTCGATGAACC3'). PCR was carried out in 25 µL containing 50 mM KCl, 10 mM Tris (pH 8.0), 0.7 mM $MgCl_2$, 100 µM dNTPs, 1U *Taq* polymerase, and 10 ng of DNA template. Samples were then subjected to one cycle at 94°C for 5 min, followed by 40 cycles at 94°C for 1 min, 55°C for 1 min, 72°C for 1 min, and a final cycle at 72°C for 8 min to complete the elongation of the PCR intermediate products. These products were characterized by electrophoresis on 2% agarose gels and stained in 0.5 µg/mL of ethidium bromide.

PCR products were sequenced directly on an Applied Biosystems 373A automated DNA sequencer (Perkin Elmer, Foster City, CA). Samples that gave a single band on agarose gels were purified (Wizard PCR Preps, Promega, Madison, WI) to remove excess primers and nucleotides. Sequencing was done with a PRISM dye terminator cycle sequencing kit (Perkin Elmer), following the manufacturer's instructions.

### Statistical Analysis

Sensitivity and specificity of the PCR-SSCP method were determined by using the test for 2X2 contingency tables. Differences in the mean MIC logs among strains with specific mutations were calculated by the two-sample Wilcoxon rank-sum test (Mann-Whitney U test).

### Results

### Rifr Pattern among *M. tuberculosis* Isolates

The 35 RIFr isolates had MIC values as follows: two isolates had an MIC of 2 µg/mL; six of 8 µg/mL; one of 16 µg/mL; one 32 µg/mL; two of 64 µg/mL; two of 128 µg/mL; eight of 256 µg/mL; one of 512 µg/mL; two of 1,024 µg/mL, and ten of 2,048 µg/mL. All 11 rifampin-susceptible isolates had MIC values $\leq$0.5 µg/mL, but all of them were resistant to at least one other primary antituberculosis agent.

### SSCP Analysis

SSCP assays were repeated at least five times with three different amplicons for all isolates with 100% reproducibility. On the basis of the SSCP results, the 35 RIFr isolates were grouped in two main categories: group one, 24 isolates (68.6%) with an SSCP identical to that of the control strain H37Rv, and group two, 11 isolates (31.4%) with an SSCP different from that of H37Rv. The MICs were variable in group one. In group two, four polymorphisms were observed with different MICs (Figure). The 11 RIFs isolates showed an SSCP identical to that of H37Rv. Therefore, the overall sensitivity of the assay was 31.4%, with a specificity of 100%. It was not possible to correlate the MIC values with the polymorphisms because each strain had a different MIC.

### DNA-Sequencing Analysis

No mutations were found in the core region of the *rpo*B gene in the 11 RIFs isolates. All 35 RIFr isolates showed a mutation by sequence analysis. Seven different missense mutations were observed, with all but one detected within the core region. These mutations produced 13 changes in amino acid content (Table). Mutations at specific codons were associated with the level of resistance; significantly higher MICs were observed when point mutations occurred in codon 513 (median MIC 2,048 µg/mL; p=0.001), in codon 526 (median MIC 2048 µg/mL, p=0.002), and in codon 531 (median MIC 256 µg/mL, p=0.002), compared with mutations at codon 516 (median MIC 8 µg/mL). Single strains

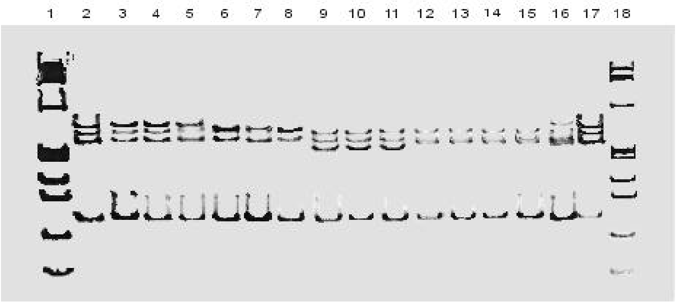

Figure. Representative polymerase chain reaction single-stranded conformational polymorphism (SSCP) patterns of rifampin resistance in *Mycobacterium tuberculosis* strains. Lanes: 1, 18, molecular weight ΦX174/*Hae*III; lanes: 2, 17, *M. tuberculosis* rifampin-susceptible control strain H37Rv; lanes 3, 4, 5 (MICs 2,8,64 µg/mL), pattern 1 rifampin-resistant strains with a pattern indistinguishable from that of *M. tuberculosis* H37Rv; lanes 6,7,8 (MICs 2, 8, 2,048 µg/mL), pattern 2a; lanes 9,10,11 (MICs 2048, 256, 256 µg/mL), pattern 2b; lanes 12,13,14,15 (MICs 2048, 2048, 256, 1,024 µg/mL), pattern 2c; lane 16 (MIC 128 µg/mL), pattern 2d.

with low-level resistance had mutations at codons 522 (MIC 8 µg/mL), 533 (MIC 2 µg/mL), and 572 (MIC 2 µg/mL) (Table). Three mutations in codon 531 had not been described previously. Neither insertions nor deletions were detected in this group of strains.

### Discussion

PCR-SSCP has been used extensively to search for genetic diseases (14,15) and recently to detect missense mutations associated with antibiotic resistance in *M. tuberculosis* (9,10,16,17). In spite of extensive and comprehensive standardization of the PCR-SSCP method, our data show that this procedure was highly specific but had poor sensitivity for detecting mutations in the *rpo*B gene in rifampin-resistant clinical isolates of *M. tuberculosis*, since two thirds of the resistant isolates had a PCR-SSCP pattern similar to that of the *M. tuberculosis* susceptible control strain H37Rv.

Our results differ from those of the investigators who first tested this technique to detect rifampin resistance in *M. tuberculosis* and demonstrated a clear association between *rpo*B mutations and the resistance profile (9). However, in recent studies, this method has detected silent and missense mutations in susceptible strains (18). We did not find these types of mutations, but we did find 24/35 (68.6%) false-negative results. Furthermore, in a recent study by Lee et al. in Korea, false-negative results were obtained in 17 (25.4%) of 67 strains (10). Although suboptimal technical conditions may account for the poor PCR-SSCP performance, missense mutations were found after nucleotide sequencing in rifampin-resistant strains.

Although the method was fully reproducible, after the data were controlled for all variables, our data also indicate that this method may perform poorly in detecting mutations in this region. To confirm our findings with PCR-SSCP, we determined the nucleotide sequence in 35 rifampin-resistant *M. tuberculosis* strains and observed missense mutations in all of them (Table).

After sequence analysis, all the highly rifampin-resistant (≥128 µg/mL) isolates were found to have point mutations in codons 513, 526, and 531, which were the most common in our study population, corroborating that these mutations are the most prevalent worldwide (19). Since our strains showed different types of mutations, we do not endorse the recently suggested idea of a geographic distribution of single mutations (20,21). Additionally, we observed three alleles in codon 531 that had not been described previously: two strains showed an TCG/GCG (Ser/Ala) exchange, one an TCG/CCG (Ser/Pro) exchange, and another an TCG/TTC (Ser/Phe) exchange.

There was a strong correlation between the degree of resistance and any nucleotide substitution in specific codons. Mutations associated with nucleotide replacements in codons 513, 526, and 531 were associated with high-level rifampin resistance, whereas mutations in codon 516 were observed in low-level rifampin resistance ($p<0.005$) (Table). Other authors have reported high (22,23) and low (24) levels of resistance associated with specific nucleotide replacements. These differences reflect the complex and crucial interaction between the drug and its target at the molecular level, where the position of the affected allele seems to be critical.

An additional interesting finding was that one strain with an MIC of 2 µg/mL did not show a mutation within the 81-bp core region, although a missense mutation ATC/TTC (Ile/Phe) was seen in codon 572, which may explain the rifampin resistance detected in this strain. This observation confirms a recent report of a rifampin-resistant strain identified in Australia with an identical mutation outside the core region of *rpo*B (25).

Although the SSCP method performed poorly in our setting, other DNA sequence-based approaches to detecting rifampin resistance may still have merit, since the vast majority of mutations occur in precisely defined positions. For example, INNO-LIPA (26) is a promising method based on specific detection of previously identified resistance-conferring mutations. Alternatively, other methods based on cell viability, such as use of mycobacteriophages and reporter genes, have shown promise (27, 28).

In conclusion, the practical implications of our study are that the PCR-SSCP method may not be a reliable tool for the detection of resistance to rifampin in *M. tuberculosis*. However,

Table. Mutations of the *rpo*B gene found in 35 rifampin-resistant *Mycobacterium tuberculosis* isolates

| Mutated *rpo*B codon | Specific Mutation | Strain n | MIC (µg/mL) | p[c] |
|---|---|---|---|---|
| 513 | CAA/AAA(Gln/Lys) | 1 | 2,048 | 0.01 |
| | CAA/AAA(Gln/Lys) | 1 | 256 | 0.01 |
| | CAA/CCA(Gln/Pro) | 1 | 2,048 | 0.01 |
| 516 | GAC/GTC(Asp/Val) | 5 | 37,118 | 0.01, 0.002 |
| 526 | CAC/TAC(His/Tyr) | 1 | 256 | 0.01, 0.002 |
| | CAC/TAC(His/Tyr) | 1 | 1,024 | 0.01, 0.002 |
| | CAC/TAC(His/Tyr) | 3 | 2,048 | 0.01, 0.002 |
| | CAC/GAC(His/Asp) | 2 | 256 | 0.01, 0.002 |
| | CAC/GAC(His/Asp) | 1 | 128 | 0.01, 0.002 |
| | CAC/GAC(His/Asp) | 1 | 2,048 | 0.01, 0.002 |
| 531 | TCG/TTG(Ser/Leu) | 1 | 32 | 0.002 |
| | TCG/TTG(Ser/Leu) | 1 | 64 | 0.002 |
| | TCG/TTG(Ser/Leu) | 4 | 256 | 0.002 |
| | TCG/TTG(Ser/Leu) | 1 | 1,024 | 0.002 |
| | TCG/TTG(Ser/Leu) | 3 | 2,048 | 0.002 |
| | TCG/CCG(Ser/Pro)[b] | 1 | 8 | 0.002 |
| | TCG/GCG(Ser/Ala)[b] | 1 | 64 | 0.002 |
| | TCG/GCG(Ser/Ala)[b] | 1 | 128 | 0.002 |
| | TCG/TGG(Ser/Trp) | 1 | 512 | 0.002 |
| | TCG/TTC (Ser/Phe)[b] | 1 | 2,048 | 0.002 |
| 522 | TCG/TTG (Ser/Leu) | 1 | 8 | |
| 533 | CTG/CCG (Leu/Pro) | 1 | 2 | |
| 572 | ATC/TTC (Ile/Phe)[a] | 1 | 2 | |

[a] This mutation is located outside the core region.
[b] Not previously described.
[c] Mann-Whitney test.

if our observation of a strong correlation between specific mutations and the level of resistance is confirmed in other settings, the level of rifampin resistance may be predictable by DNA sequence-based resistance detection methods.

### Acknowledgments

We thank B. Chavez-Mazari and G. Hernandez for technical support.

This study was partially supported by grants from CONACyT, G-26264/M, and the Fogarty International Center, FIRCA, PA-95-011, NIH, ICIDR-5U01AI35969 and ERID-TW-96-001 and UNAM-PADEP (201313). Miriam Bobadilla-del-Valle was a recipient of a CONACyT scholarship (No. 118152).

Dr. M. Bobadilla-del-Valle is a molecular microbiologist in the Laboratory of Clinical Microbiology, Instituto Nacional de Ciencias Médicas y Nutrición Salvador Zubiran, Mexico. Her research involves the molecular basis of drug resistance in tuberculosis.

### References

1. Pablos-Mendez A, Raviglione MC, Laszlo A, Binkin N, Rieder HL, Bustreo F, et al. Global surveillance for antituberculosis-drug resistance, 1994-1997. N Engl J Med 1998;338:1641-9.
2. World Health Organization/IUATLD Global Project on Anti-Tuberculosis Drug Resistance Surveillance. Anti-tuberculosis drug resistance in the world. Geneva: The Organization; 1998. Pub. no. WHO/TB/97.229.
3. Sifuentes-Osornio J, Ponce-de-Leon A, Camacho-Mezquita FE, Bobadilla-del-Valle M, Infante-Suarez ML, Ramirez-Fernandez N, et al. Resistance of *Mycobacterium tuberculosis* in Mexican patients. I. Clinical features and risk factors. [Resistencia de *Mycobacterium tuberculosis* en pacientes mexicanos. I. Caracteristicas clinicas y factores de riesgo]. Rev Invest Clin 1995;47:273-81.
4. Kato-Maeda M, Sifuentes-Osornio J, Bobadilla-del-Valle M, Ruiz-Palacios GM, Ponce-de-Leon A. Drug resistance among acid-fast bacilli. Lancet 1999;353:1709.
5. Garcia-Garcia ML, Jimenez-Corona ME, Ponce-de-Leon A, Jimenez-Corona A, Palacios-Martinez M, Balandrano-Campos S, et al. *Mycobacterium tuberculosis* drug resistance in a suburban community in southern Mexico. Int J Tuberc Lung Dis 2000;4 (Suppl 2):168-70.
6. Granich RM, Balandrano S, Santaella AJ, Binkin NJ, Castro KG, Marquez-Fiol A, et al. Survey of drug resistance of *Mycobacterium tuberculosis* in 3 Mexican states, 1997. Arch Intern Med 2000;160:639-44.
7. Frieden TR, Sherman LF, Maw KL, Fujiwara PI, Crawford JT, Nivin B, et al. A multi-institutional outbreak of highly drug-resistant tuberculosis. JAMA 1996;276:1229-35.
8. Garcia-Garcia Ml, Ponce-de-Leon A, Jimenez-Corona ME, Jimenez-Corona A, Palacios-Martinez M, Balandrano-Campos S, et al. Clinical consequences and transmissibility of drug-resistant tuberculosis in southern Mexico. Arch Intern Med 2000;160:630-6.
9. Telenti A, Imboden P, Marchesi F, Lowrie D, Cole S, Colston M, et al. Detection of rifampin-resistance mutations in *Mycobacterium tuberculosis*. Lancet 1993;341:647-50.
10. Lee H, Cho SN, Bang HE, Lee JH, Bae GH, Kim SJ, et al. Molecular analysis of rifampin-resistant *Mycobacterium tuberculosis* isolated from Korea by polymerase chain reaction-single strand conformation polymorphism sequence analysis. Int J Tuberc Lung Dis 1998;2:585-9.
11. Metchock BG, Nolte FS, Wallace R Jr. *Mycobacterium*. In: Murray P, Baron E, Pfaller M, Tenover F, Yolken R, editors. Manual of clinical microbiology. Washington: ASM Press; 1999. p. 399-437.
12. Lee CH, Heifets L. Determination of minimal inhibitory concentrations of antituberculosis drugs by radiometric and conventional methods. Am Rev Respir Dis 1987;136:349-52.
13. Wilson K. Preparation of genomic DNA from bacteria. In: Ausbel FM, Brent R, Kingston RE, Moore DD, Seidman JG, Smith JA, et al., editors. Current protocols in molecular biology. Vol. 1, New York: Green and Wiley-Interscience; 1990. p 2.4.1-2.
14. Orita M, Iwahana H, Kanazawa H, Hayashi K, Sekiya T. Detection of polymorphisms of human DNA by gel electrophoresis single-strand conformation polymorphisms. Proc Natl Acad Sci U S A 1989;86:2766-70.
15. Susuki Y, Orita M, Shiraishi M, Hayashi K, Sekiya T. Detection of *ras* gene mutations in human lung cancers by single-strand conformation polymorphisms analysis of polymerase chain reaction products. Oncogene 1990;5:1037-43.
16. Kim BJ, Kim SY, Park BH, Lyu MA, Park IK, Bai GH, et al. Mutations in the *rpo*B gene of *Mycobacterium tuberculosis* that interfere with PCR-single strand conformation polymorphism analysis for rifampin susceptibility testing. J Clin Microbiol 1997;35:492-4.
17. Telenti A, Honoré N, Bernasconi C, March J, Ortega A, Heym B, et al. Genotypic assessment of isoniazid and rifampin resistance in *Mycobacterium tuberculosis*: a blind study at reference laboratory level. J Clin Microbiol 1997;35:719-23.
18. Telenti A, Imboden P, Marchesi F, Schmidheini T, Bodmer T. Direct automated detection of rifampin-resistant *Mycobacterium tuberculosis* by polymerase chain reaction and single-strand conformation polymorphism analysis. Antimicrob Agents Chemother 1993;37:2054-58.
19. Ramaswamy S, Musser JM. Molecular genetic basis of antimicrobial agent resistance in *Mycobacterium tuberculosis:* 1998 update. Tuber Lung Dis 1998;79:3-29.
20. Schilke K, Weyer K, Bretzei G, Amthor B, Brandt J, Sticht V, et al. Universal pattern of *rpo*B gene mutations among multi-drug resistant isolates of *Mycobacterium tuberculosis* complex from Africa. Int J Tuberc Lung Dis 1999;3:620-6.
21. Kapur V, Li L, Iordanescu S, Hamrick MR, Wagner A, Kreiswirth B, et al. Characterization by automated DNA sequencing of mutations in the gene (rpoB) encoding the RNA polymerase b subunit in rifampin-resistant *Mycobacterium tuberculosis* strains from New York and Texas. J Clin Microbiol 1994;32:1095-8.
22. Moghazeh S, Pan X, Arain T, Kendall C, Musser J. Comparative antimycobacterial activities of rifampin, rifapentine and KRM-1648 against a collection of rifampin-resistant *Mycobacterium tuberculosis* isolates with known *rpo*B mutations. Antimicrob Agents Chemother 1996;40:2655-7.
23. Williams DL, Spring L, Collins L, Miller LP, Heifets LB, Gangadharam PRJ, et al. Contribution of *rpo*B mutations to development of rifamycin cross-resistance in *Mycobacterium tuberculosis* Antimicrob Agents Chemother 1998;42:1853-57.
24. Bodmer T, Zurcher G, Imboden P, Telenti A. Mutation position and type of substitution in the ß-subunit of the RNA polymerase influence in-vitro activity of rifamycins in rifampicin resistant *Mycobacterium tuberculosis*. J Antimicrob Chemother 1995;35:345-8.
25. Lilly K, Yuen W, Leslie D, Cole PJ. Bacteriological and molecular analysis of rifampin-resistant *Mycobacterium tuberculosis* strains isolated in Australia. J Clin Microbiol 1999;37:3844-50.
26. Hirano K, Abe C, Takahashi M. Mutations in the rpoB gene of rifampin-resistant *Mycobacterium tuberculosis* strains isolated mostly in Asian countries and their rapid detection by line probe assay. J Clin Microbiol 1999;37:2663-6.
27. Jacobs WR Jr, Barletta RG, Udani R. Rapid assessment of drug susceptibilities by means by luciferase reporter phages. Science 1993;260:819-22.
28. Banaiee N, Bobadilla-del-Valle M, Bardarov S, Riska PF, Small PM, Ponce-de-Leon A, et al. Evaluation of luciferase reporter mycobacteriophage technology for detection, identification, and antibiotic susceptibility testing of *Mycobacterium tuberculosis* complex in Mexico. Abstracts and final program of the 2001 Keystone Symposia on Molecular and Cellular Aspects of Tuberculosis Research in the Post Genome Era (B1). Taos, New Mexico. January 25-30, 2001. Abstract 204, p. 69.
